# Multiple myeloma in people of working age in Czechia, Germany, and Poland: findings from a qualitative interview study

**DOI:** 10.1007/s11764-023-01510-1

**Published:** 2023-12-20

**Authors:** Liz Forbat

**Affiliations:** University of Stirling, Stirling, UK

**Keywords:** Myeloma, Work, Discrimination, Disclosing diagnosis, Support

## Abstract

**Purpose:**

The study sought to understand the experiences of working age adults with myeloma and their partner/family members, living in Czechia, Germany, and Poland.

**Methods:**

Qualitative interviews were conducted with 36 working age adults living with myeloma, and three family members. Data were collected from May to October 2022. Thematic analysis was applied to the data.

**Results:**

Healthcare and state support within each country are described. The degree of work engagement was informed by patients’ symptom burden, treatment needs, state financial aid, and family/financial obligations. Many did not conceptualise their status as involving ‘return to work’ as they had continued to be engaged with their jobs throughout. For some, remote working enabled them to manage treatments/side-effects and their job, while avoiding infection. In some cases, patients did not tell their employer or colleagues about their illness, for fear of discrimination.

**Conclusion:**

While experiences varied between countries, common across accounts was a struggle to balance ongoing treatments with employment, at a time when participants were expected to finance their own households and maintain their income and roles.

Implications for Cancer Survivors

To improve quality of life, clinical discussions around treatment decision-making should take into account patients’ attitudes/approach to work, type of work engaged in, and other activities considered important to them. European Union and national cancer plans should set out optimum standards for employers, to ensure an equitable benchmark for how employees are supported. Such approaches would improve legal protections and better enforcement of employer policies to accommodate patients’ limitations in the workplace.

## Introduction

Multiple myeloma (hereafter, *myeloma*) is a rare blood cancer. Although myeloma is incurable, treatments can significantly lengthen survival and improve quality of life. Survival has improved, particularly in working-age people [1]. EU27 data show age-standardised mortality in 2020 of 6.2 per 100,000 and 3.9 for men and women respectively [2]. Five-year survival rates are approximately 50%, rising to 60% in people aged 65 and under [3]. Increased incidence and longer survival intervals therefore point to a need to understand myeloma patients’ experiences of work [4].

Since the median age of diagnosis is 66–70 years, and 37% are younger than 65, many patients are of working age[5, 6]. Morbidity over prolonged timeframes means people often live with high symptom burden including pain, fatigue, and anxiety [7–9].

The impact of physical symptoms [10, 11], psychosocial factors [12, 13], and finances [14, 15] on return to work and employment have been well described across different cancer types. Fatigue is cited as a core symptom of concern to cancer patients wishing to remain in employment [16, 17]. Workplace adjustments are recognised as important in facilitating people’s return to work [18], alongside managing stigma and discrimination [19]. While younger people are more likely to return to work, this may require a reduction in working hours due to symptoms [20] or stigma [21], which can risk financial insecurity [22]. Where people lack symptom control and workplace support, then engagement with work is, unsurprisingly, impoverished [23]. Yet, engaging in work has shown to result in more happiness for (male) cancer survivors living with their disease [24] and hence can be important psychosocially as well as financially.

A recent systematic review identified 34 papers on employment and working age people with myeloma, which reported core themes on side-effects, stigma, and medicines; relationships; identity; and privilege and income [25]. The review noted a need to deepen understandings of work and myeloma to inform policy and practice. The combination of illness, treatment, and work and life-cycle transitions occurring in working age adults present specific stressors. Being of working age also presents opportunities for some, such as employment-funded health insurance, paid sick leave, and a non-illness focus for life. These impacts require better understanding of the complexities of employer/colleague support, patient engagement in meaningful activity, and balancing work with illness.

The current paper aims to add to the limited evidence base of people with myeloma engaging in work. The primary research question was: What it is like for working-age adults to live with myeloma? This paper describes a sub-set of data, focused on engagement in work and other important activities.

## Methods and design

This was an interpretative qualitative interview study.

### Setting

Three countries — Czechia, Germany, and Poland — were chosen to allow for diversity in sociocultural context, and its impact on social security, work, and health services. The choice of countries also sought to privilege nations not traditionally represented in the evidence, noting that a recent systematic review only identified one paper with German participants, and none from Czechia or Poland [25]. Age-standardised incidence of myeloma in these countries are 5.4 (Czechia), 7.6 (Germany), and 5.8 (Poland) [26].

### Sample and recruitment

Study inclusion criteria were:

(i) Being a working-age adult living with myeloma,
(ii) Having undergone at least one line of treatment for myeloma, or be a family member of someone having received at least one line of treatment,
(iii) Residing in one of the three countries,
(iv) Willing to provide informed consent.

As ‘working age’ is a concept that varies by country, genders, and professions, the country’s official retirement age was adopted as the upper limit, with 18 as the lower age limit. OECD data [27] shows the standard retirement age at the time of recruiting to the study (2022) were:

1. Czechia: 63 years for men; 62 years 4 months for women,
2. Germany: 65 years and 9 or 10 months (depending on year of birth),
3. Poland: 65 years for men; 60 years for women.

A minimum sample size of 12 patients per country was sought, informed by principles of theoretical sufficiency. As far as possible, with a rare illness, the sample aimed to be heterogenous.

Recruitment proceeded through Myeloma Patients Europe’s (MPE) established advocacy and patient support networks using emails and newsletters. Adverts for the study were also posted in social media channels. Snowball methods were also employed to encourage recruitment from participants’ networks.

### Data collection

Interviews were conducted between May and October 2022 using online video platforms; most were conducted in a single session, but one interview was conducted in two parts on different days. Interviewees could choose whether to participate individually or with their partner; most opted for individual interviews, with one dyadic interview conducted.

Interviews were recorded and audio-transcribed verbatim. Field notes were made by researchers, which informed the coding framework and aided interpretation.

Interview questions were developed by the study team, drawing on a systematic review to identify areas which were not well described in the literature [25]. Feedback on interview questions was provided from the study steering group (which was comprised of clinicians and people with lived experience of myeloma). Questions were adapted dynamically during interviews to follow the narrative of interviewees. Interviews focused on paid employment as well as unpaid/voluntary work and other activities important to the individual for which they would need to pay someone else to conduct if they were unable. Questions focused on participants’ subjective experiences and accounts of being working age, living with myeloma.

Interviews were conducted by two female researchers who hold qualitative PhDs, and hence had extensive prior qualitative research experience. One researcher was employed full time as the study research fellow and conducted most of the interviews. The other researcher worked part time on the study in the role of principal investigator and conducted one interview. Interviewers and interviewees were not known to each other prior to data collection, and the team’s motivations for conducting the research were summarised in the participant information sheets. Professional interpreters, not previously known to interviewees, were used for Czech and German interviews. Polish interviews were conducted by a Polish member of the research team. Interpreting is not a neutral or value-free enterprise [28], and interpreters play an active role in constructing both questions and responses.

### Analysis

Thematic analysis underpinned the analytic approach, following the five-step process outlined by Braun and Clarke [29]. Stage 1 involved familiarisation with the dataset through repeated re-readings, and for interviews conducted in Polish additional familiarisation via translation to English and transcription. Stage 2 involved identifying an initial thematic framework, which was used in stage 3 where data was indexed with reference to the thematic framework. In stage 4, data were synthesised from across respondents into consolidated themes. Stage 5 focused on data interpretation and finalisation of key themes generated from the data. Two researchers were involved in the analysis to generate codes, themes, and superordinate themes.

Analysis was inductive and included an intersectional approach, to create space for considering the interactions between micro- and macro-levels, focused on those most relevant to the research question and sample: culture, socio-political context, gender, and age. Respondent checking of transcripts was not used, but findings were discussed with the steering group.

Nvivo20 was used to store and organise the data, and facilitate development of codes, memos, and themes. Both interviewers were involved in coding and analysing the data.

### Ethical approvals

Ethical approval for the study was provided by the General University Ethics Panel from the University of Stirling. All participants provided written consent. To preserve respondent anonymity quotes are presented solely by country, participant code, then gender and age of the individual, for example G5, M34 (German respondent number 5, a man aged 34). Identifying details have been removed.

## Results

### Sample

Interviews were conducted with all 39 people who expressed interest in the study (see Table 1). The sample size target (*n* = 12) was exceeded in two countries, but not achieved in Germany despite extensive efforts through patient networks. The lower recruitment in Germany may be explained by patient expectations of remuneration for participation, which is not normative in Czechia or Poland. Theoretical sufficiency was nevertheless reached, within and across countries. The lower number of partners taking part meant that there is less confidence in theoretical sufficiency for this dataset, yet the findings map on to patient accounts well, lending face validity to the themes and findings. Interviews ranged from 37 to 91 min, with an average of 57 min.

**Table 1:** Participant characteristics

Country	Role		Gender		Age range (average)
	Patient	Partner	Female	Male	
Czechia (*n* = 13)	12	1	7	6	46–62 (52)
Germany (*n* = 10)	10	0	4	6	34–63 (52)
Poland (*n* = 16)	14	2	8	8	38–65 (51)
**Total: 39**	**36**	**3**	**19**	**20**	34–65 (52)

The number of treatments varied across participants. In Czechia: nine participants had received first line of treatment, three had received second line of treatment. In Germany, no-one had received a second line of treatment; six people had full/partial remission, and four people had possible relapse under observation. In Poland, ten participants had received first line of treatment, four had second line, and one person who had received two lines of treatment and was under observation for a second relapse.

### Coding tree

Three superordinate themes were derived from the analysis (Table 2). Only the first superordinate theme, with the relevant second order analysis, is reported in this paper: reconciling illness with work, to allow sufficient depth of reporting.

**Table 2:** Coding framework of the qualitative interviews

Initial codes	Second order themes	Superordinate theme
(i) State support (ii) Side-effects (iii) Treatment choices (iv) Collegial support (v) Disclosure to the workplace (vi) Finances (vii) Job security (viii) Discrimination (ix) Gender (x) COVID-19	State financial support	Reconciling illness with work
	Engaging in work	
	Disclosing the diagnosis	
	Working while ill	
	Workplace support	
(i) Family and partners (ii) Friends (iii) Disclosure (iv) Discrimination (v) COVID-19 (vi) Changes post-treatment (vii) Priorities change (viii) Awareness of dying: systemic family decisions (ix) Gender roles	Managing uncertainty	Living with a chronic, incurable disease
	Adjusting to illness	
	COVID-19	
	Relationship changes	
(i) Priorities for change (ii) Patient organisations (iii) Psychological support (iv) Communication with healthcare staff (v) Palliative care (vi) COVID-19 risks/changes in service provisions (vii) Discrimination (viii) Gender	Access to treatment and services	Systems, structures and services
	Supportive and palliative care	
	Clinical interactions	
	Patient support	

### Reconciling Illness with work

People’s private and professional lives are embedded in, and shaped by, their countries’ cultural milieu. Living with myeloma is often accompanied by a sense of precarity, with respondents having access to varied state resources and protections to support them.

### State financial support

People described their understanding of, and access to, financial support from the state, for working-age people with a serious illness. Some were not aware of their rights for state support and indicated that they received lower levels of assistance that was available.

Respondents from Czechia reported that disability allowance [invalidní důchod] was often not sufficient for a single person, and they had to rely on their partners’ income or continue working themselves. There are three tiers of state support for disability, awarded following repeated health assessments. Tier one is for those with a 35–49% decreased ability to work, tier two for people 50–69% less able to work, and tier three for people with 70% or higher inability to work. Some respondents did not apply for the disability allowance as they were in employment, even though they would still be entitled to it by law.

I’m just working regularly without any sort of disability allowance or anything. I wasn’t even applying because I don’t really think I would be entitled, because I'm fully able to work. Nothing is stopping me from working. C11, M58

Most respondents received 60–70% of their former salaries. Patients could also use a ‘disability card’ which provided different levels of support based on symptoms, such as free public transport and taking priority in queues. Many were financially impacted by cost of treatments despite state support:

For the physiotherapy, that is covered with the insurance, and for the spa physiotherapy treatment, I only had it paid once by the insurance right after the transplant. After that I have to pay it myself and it's always a three week treatment but the three weeks cost more than my free disability pension. C4, F55

Most respondents from Germany expressed their satisfaction with the financial support provided by the state, which they deemed easy to access and sufficient. They described tax reliefs and reductions for those in employment, and health insurance covering a range of health services. Some respondents mentioned tax allowances for their unpaid carers. A disability certificate [Schwerbehindertenausweis] (formal evidence of disability) was reported to be easily obtained. If a person was unable to work due to sickness, their employer was obliged to pay their salary for six weeks, with the state continuing to pay sickness benefit [Krankengeld] for up to 78 weeks (1.5 years). Overall, with minor exceptions, those treated in Germany showed trust in state regulations, felt their employers’ rights were well protected, and that there was generally enough state assistance available for them to live comfortably.

As soon as I got the diagnosis, I filled out an application for disability, and I got 100% disability for an indefinite period. G2, F52

Respondents in Poland described various regulations around sickness allowance (maximum of 182 days per year), followed by the rehabilitation leave (10 additional days of leave, for employees with disability status), rehabilitation benefit (a benefit for up to 12 months granted to those who have already used their sickness benefit but are still unable to work), and disability allowance (renta). The percentage of the salary paid and the length of the sickness allowance were variable (based on, for example, previous salary, age, or if the patient was hospitalised or pregnant when problems occurred). Respondents in the study sample received 70–90% of their former income. As it is not possible to claim two Social Insurance Institution allowances at the same time, patients must choose between renta, rehabilitation allowance, or any other state income. Some respondents continued working while claiming renta, which stipulates a legal maximum of seven hours work per day. Respondents drew on a mixture of paid sick leave, family support, and state benefits:

At first I used my sick leave, then there was a rehabilitation benefit, and then there was an unpleasant moment which we call renta [disability allowance], because I wanted it, and I did not want it … I mean, probably if I were alone, if I didn’t have a husband, it would be hard to make the transition to renta and make a living ... I got used to this life on benefits. P4, F47

Across countries, access to benefits which required repeated assessment was considered inappropriate for a chronic and relapsing disease such as myeloma. Respondents stated that although the remission periods may be long, symptoms could potentially be just as burdensome throughout. For an incurable disease, ongoing assessment was considered unduly arduous.

### Engaging in work

Twenty-six participants (including all three relatives) were employed at time of interview, with 13 unemployed and in receipt of benefits. Many had worked during treatment. Three people with myeloma described themselves as self-employed. Some engaged in voluntary work, such as for myeloma support organisations, and one continued her previous work on a voluntary basis.

Although employees’ rights are protected by law in all three countries, there were differences in the way participants expected them to be honoured by employers, which in turn affected people’s willingness to disclose their illness at work. Concerns about job security and financial precarity influenced treatment choices, with patients occasionally compromising their health to retain their jobs. Respondents spoke about their work obligations, and career paths that were temporarily or permanently disrupted by myeloma. When there was confidence that employment law and protections would be followed, patients had less disruption to their working lives and family life, leading to an all-round greater sense of security.

In Poland, people reported that the prevalence of treatment side-effects and lack of maintenance treatment made it more difficult to work. Consequently, they reported that suboptimal healthcare provision restricted engagement in employment. Such concerns were not expressed in Czechia or Germany.

Some people were eager to work as a sign that they were ‘healthy again’ [G8, F51], and minimise disruptions to their life. Engaging in work however was often not easy. Some had long-term mental health problems due to the myeloma. Some found it hard to re-connect with their colleagues or find the same motivation as before. Keeping pace with busy work schedules was difficult, although some managed to reduce their hours or adjust their schedule around treatment or fatigue.

Some interviewees worried that work stress could make their health deteriorate. Other respondents found that they could not continue in their old industry or perform work that was too physically or emotionally demanding. These people switched professions, reduced their hours to part-time, or ceased working:

I came back part-time. And after one month I already knew that my body could not cope (…) so the decision had to be made, well, whether it’s work, or are we fighting for this life that was saved, so not to ruin all this. Because every infection brings us closer, this is how my doctor explained this to me at the time, that in myeloma every infection brings us closer to the relapse of the disease. So, I moved, I quit. P4, F47

A reduction in working hours was a common feature of both patients and partners, sometimes by choice:

I don’t generally work as much as I used to and I just don’t think I am as dedicated, as engaged with things as I used to be. G1, M49

For others, exiting employment was not by choice. One respondent lost their job due to not being able to manage the work, alongside additional tasks and ongoing symptom burden:

I changed the job because my employer wasn’t happy that I was ill. After the illness, unfortunately, my strength weakened a little, I also got new tasks at work, which unfortunately I couldn’t cope with. And I refused to accept additional tasks and therefore I was let go. P14, F57

For self-employed people, there were considerable risks and worries associated with managing symptoms and considering return to work; one respondent had felt mis-led about his recovery from treatment, which left him in considerable debt:

[The doctors] said, ‘Oh, no problem at all. After the stem cell transplantation, you will be almost your old self. You will be able to do everything you’ve been doing before.’ Which obviously was an outright lie. I trusted them at the time, so ordered new machines, about €200,000-worth of investment. Then I came to [the hospital] for the stem cell transplantation, and the first appointment with the doctors there, within five minutes it was obvious that everything I’ve been told before was completely wrong, but there was no way back (…) the machines had already been delivered. G10, M60

### Disclosing the diagnosis at work

In some cases, patients did not tell their employer or colleagues about their illness. Some treated their illness as personal information, of no relevance to others, while others had justifiable reservations about the potential workplace discrimination:

One [firm made a job offer] and when they learnt I have this disease, they told me not to join. P3, M38

Such discrimination, albeit technically illegal, could be difficult to prove. Discrimination also led to exclusion of patients with chronic diseases from the job market; as Polish respondents reflected, there is not enough retraining support for those over the age of 40. Overall, however, respondents felt that their job was secure, either due to the good relationship with their employer, or state legal protections for those classified as disabled or with a long-term illness:

In Germany (…) You can’t just fire disabled people with a [disability] certificate. Yes. That’s not possible (…) The legislation works in Germany that way. G5, M34

Some shared as little as they thought was necessary, not making their disease a secret, but not sharing too many details either. Those who decided to share more information mentioned practical reasons such as notifying their employers of their work availability and giving them notice for when they need to attend medical appointments:

They do know, in general, however it’s very superficial knowledge. I do not involve them in any details, I don’t explain exactly what I suffer from, they do know it’s some form of chronic disease. P10, M50

Many respondents who worked remotely did not feel closeness with their work colleagues, and consequently did not share any details regarding their health. Those who did not trust their employers decided not to tell anyone, and ensured their formal paperwork about the illness avoided mentioning the specific diagnosis:

I rather would not tell them. I made an online check whether to tell or not to tell. The result for me was rather not to tell the employers. [In my] certificate of disability, [myeloma specifically] is not mentioned. G4, M53

Although many respondents said they did not want to overwhelm their colleagues with cancer stories and cause any hostility, they observed some reverberations in their workplaces. In one case, the respondent’s diagnosis prompted her colleagues to rush for their own (previously neglected) health check-ups:

Yes, everyone knew I was ill. Everyone knew. When they found out that I was ill, everyone went to get tested right away, everyone had a blood test [laugh]. P9, F58

Some self-employed interviewees struggled to articulate the impact of myeloma, and explored State support:

I would like to discuss with my doctor the possibility of the disability allowance. (…) There are times when I think [my business partner is] not yet able to fully understand the disease and the seriousness of the situation, but it’s been getting better, and I think it’s on quite a good level right now. (…) We look at it as he helps me now and then if he needs help then I will be prepared to do it for him. C8, M52

### Working while ill

Treatment side-effects caused memory issues and concentration problems for some; others found they experienced fatigue, could not stand for long, or had to frequently rest, all of which impacted their employment. Other patients experienced spontaneous bone fractures, which for those with physically demanding work, effectively excluded them from their previous professions. The most cited limitation was neuropathy:

I just couldn’t physically go to work for eight hours anymore. I can’t do it anymore and, paradoxically, it wasn’t because of the illness itself, but because of the treatment. It has caused health complications that make it impossible for me to work full time (…) some of the symptoms that are caused by the initial treatment are irreversible. For example, neuropathy in my legs or feet. C1, F58

Treatments also caused insomnia, triggered early menopause, and created many symptoms difficult to manage while working. Steroids were a particular burden, with the energy fluctuations, and mood disturbance making interpersonal interactions problematic:

Taking steroids, that’s a heavy topic, because they cause such an emotional swing of moods and energies. At the time of taking a steroid we feel as if we are high for about a day and a half, like on some very high drug, we experience multitasking, we can be a ‘multitasker’ who does, who tells the whole family what he thinks about them – one should not make [laugh] any decisions during that time, yes, neither divorce ones nor any other [decision]. P9, F58

Some tried to work because of financial worries. Working through necessity was especially the case for women. Nine women said that their disability benefits would not be sufficient for them to live on, and they relied on their partner’s salary or had to work themselves:

I'm very lucky that my husband is able to earn enough for the both of us, so I’m not in a situation where I have to work. (…) Yes, I get what’s called a disability pension, but if I was by myself, it wouldn’t be enough to just live on this. C1, F58

Those on temporary work visas were not entitled to state protection and had to work to retain their residency rights and access to healthcare:

I had to keep working because I am a foreigner here, I am not a citizen here. If I stopped working my insurance goes away and I will be soon asked to leave the country. P3, M38

Some respondents worked because of financial necessity, even though they saw their work conditions as hazardous for their health:

I had to quit my job, and the reason was that I worked in a care home for elderly people and there’s frequent illnesses, infections going around. (…) I was off sick for a year-and-a-half and I was trying my best to avoid people to not contract COVID (…) So I have been given a disability pension but I work part-time in my old job in the care home, just because the financial situation was just disastrous, so I had to go to work. C5, M50

Thus, many continued to work despite not necessarily being well enough to do so.

### Workplace support

Most respondents reported receiving support from their employers. Some larger companies had formalised support mechanisms, occasionally more generous than the law required. In other cases, colleagues stepped in with informal help, for example, organising fundraisers. Some employers tried to shield their ill employees from COVID-19 and extended their right to remote work when the rest of the staff had returned to the workplace. Some respondents said they were assured they could take time off for treatments and medical appointments, work from home, or move to part-time work:

I did inform my employer (…) and he’s been very understanding of it. He gave me all the time needed for going to therapy, for doing my health checks, so formally I was still working (…). But I did get my time to do all my health check-ups (…). Then in December I had to do the stem cell treatment; I took six months' sick pay (…) I am back at work since July on a full-time basis, but in agreement with my employer that I will still need extra time for my treatments and therapies. G1, M49

Flexible working was particularly important for those experiencing side-effects of maintenance drugs, such as diarrhoea or fatigue. In other cases, work tasks were limited due to occupational health and safety, for example, working on offshore turbines. What was particularly important for those in treatment was reassurance that their job would be waiting for them; this was often attributed to individual good will and good personal relations and not legal protections, even though these should technically be in place:

There was no problem with our management…we have a good relationship with each other, so [that wasn’t an issue]. Well, you can even say that they got worried that I was ill. P11, M47

Some respondents were disappointed with the lack of support from their colleagues. COVID-19 did however mean that pandemic adjustments allowed them to feel better supported due to the more flexible nature of work and being able to rest when they needed.

For self-employed interviewees, there were different stressors and benefits for workplace support. Several relied on their business partner, while others ran a family business with concomitant reliance more on those relatives:

I am a self-employed person in the farming industry, and so I’m sort of independent, self-employed person. (…) I worked full-time before the treatment, and I continue to do so. (…) I do my self-employed work with my brother, and we're partners. During the transplant, and afterwards, he had to work twice, he had to do my work as well. C6, 49

## Discussion

This is the first study to bring together the experience of myeloma patients from three mainland, often-understudied, European countries: Czechia, Germany, and Poland. It addresses a call to conduct more cross-country studies in employment and cancer, to reduce inequalities [30] and share best practice from different cultural and legal environments. The European Code of Cancer highlights the need for employers to address employment-related issues [31], not least because of the financial impact of cancer on loss of productivity [32]. These findings highlight some of the specific difficulties experienced by people living with myeloma.

The impact of cancer on people’s ability to engage in work is well-recognised [33, 34]. Limiting fatigue [35] may be necessary, but is insufficient to support affirming engagement in work. Disclosure of a cancer diagnosis at work is a complex social phenomenon; patient concerns with being stigmatised, deemed unable to perform adequately, and managing confidentiality has been noted in the literature[33]. Yet few cancers have the relapsing remitting pattern of myeloma, which positions this patient cohort as managing a different set of concerns regarding their long-term survival and morbidity. While understandable, the hesitance in sharing the diagnosis with managers and colleagues reduces the potential supports from those systems, which are already recognised as inadequate[36].

The dominant framing in the literature of ‘return to work’ is a misnomer for many of our study’s cohort. Working while unwell, and managing treatments and side-effects alongside employment, was common. Hence, ‘return’ to work was not how they framed their experiences. Other studies have begun to trouble the nomenclature [37], but still view ‘return’ as a routine rather than anomaly. Ensuring that patient and employer information and language are nuanced and fit the relapsing/remitting course of illness and treatments will be important if there is to be alignment between patient needs and employers’ policies.

There is a need for employment policies and support mechanisms to reflect the unique prognosis, symptom profile, and financial commitments of working age people with myeloma. International organisations and policy advocates should adopt a pan-EU strategy on supporting patients’ rights and expectations in cancer survivorship, aligning with the European cancer plan [38]. While national policy and employers approaches will be retained by local administrations, agreeing international standards would be a positive step toward supporting working age adults with this condition. The impact is likely to be felt more intensely in countries such as Czechia and Poland which have limited return-to-work policies [39]. Without such advocacy and change, the risks of financial toxicity increase considerably as people cannot continue to work, or afford co-pays on insurance policy [22, 40]. Discussing finances and employment also needs to be integrated into clinical discussions, and rendered explicit in treatment decision-making [41], which will benefit those who are employed and those in a more precarious situation, with longer working hours and reduced sick leave use, of those who are self-employed [42]. Adjusting employment policies would also embed recognition of the importance of work to people’s identities [25].

Enshrining and honouring protections for disabled people will be important in changing the culture of hesitancy in disclosing diagnoses for fear of repercussions. People of working age occupy a unique position, with the potential for a decade or more working years ahead of them, yet stigma and threat of dismissal prevent open discussion. By not sharing details of the condition, opportunities are lost for direct support of the person with the condition, and for educating people about this rare cancer, and tackling cancer myths[43].

### Limitations

Country-specific recruitment was low for Germany with ten participants. Despite the smaller sample size, theoretical sufficiency was achieved, and the whole data corpus demonstrates some compelling and repeated concerns expressed by people living with myeloma. The findings, while not necessarily representative of all patients’ experiences, demonstrate clear areas for improvement in policy and practice.

Very few relatives/unpaid carers were recruited to the interview study. The limited accounts of carers in both literature and interviews represent an important absence in the evidence [44], meaning that recommendations are based primarily on patient perspectives of their own needs and speculation about the wider family/relational system’s needs.

Interviewees were a self-selected sample of people willing to talk about their experiences, who were often engaged in patient organisations, and internet savvy. Consequently, their accounts may not include the broadest potential range of experiences. Despite ongoing recruitment for several months, recruitment in Germany was lower than in the other countries.

We sought to interview people in their first language, to provide respondents with the most accessible form of conveying their experience. However, in working with interpreters, there is the potential for some nuance to have been conveyed in a way the respondent had not intended [45, 46]. Further, some interviewees wished to be interviewed in English or Polish even when that was not their first language, which may have constrained fully articulating their experiences.

## Conclusions and recommendations

Clinical discussions around treatment decision-making should take into account patients’ attitudes/approach to work, type of work engaged in, and other activities considered important to them. This approach will enable patients to achieve better quality-of-life and avoid financial toxicity, or dependence on family and state. The approach will require treating teams, patients, and employers to work more closely and explicitly around the treatment goals and choices. This may, for example, lead to more flexible approaches to treatment modalities, or work routine, to reduce the impact on the patient. Such an approach may require involvement of health insurance providers, and hence involvement of additional systems and organisations.

Interventions by employers can play an important role in supporting people to continue working. Stigma is a modifiable variable which can aid people’s ability to successfully engage in work. Addressing stigma [47] and discrimination [48] together would improve patients’ lives and reduce concern that disclosing their diagnosis would lead to unfavourable treatment in the workplace. Multi-disciplinary teams treating myeloma patients should include access to advice on holistic cancer care issues, returning to work and work-place issues (such as occupational health specialists trained on this topic).

Legal protections and better enforcement of employer policies to accommodate patients’ limitations in the workplace would be beneficial. Such adaptations would alleviate feelings of employee inadequacy and prevent internalising stigma, which can impair their performance and/or willingness to remain in employment. Workplace adjustments such as remote or flexible working (whenever possible) could help patients manage their symptoms, and mitigate fears of potential job loss leading to worsening financial toxicity and relational strain.

European Union and national cancer plans should set out optimum standards for employers, to ensure patients, particularly those of working ages, are able to access advice and support on returning to work, finances, and holistic issues associated with their cancer

Cancer/health policy and regulations should consider the needs of people who have incurable, relapsing and remitting, cancers. Specific attention should include the protections and supports where patients and unpaid carers may leave and return to work several times, as their needs will differ across the trajectory of the illness and change over time.

Research could help develop greater understandings of how identity is shaped by employers, colleagues, and customers. Further, examining such relational networks could aid strategies which seek to improve awareness of myeloma in the community. While myeloma remains a rare disease, there is great capacity for employees to share knowledge and understanding of this cancer impacting their own identity as well as colleagues’ insights into illness.

The experiences of unpaid carers/family members employment is an under-researched area. In the absence of primary research data, practice and policy changes risk being based on patient perspectives, rather than carers’/relatives’ views and experiences.

## Data Availability

The data from this study are available from the corresponding author upon request and approval by Myeloma Patients Europe, and provision of ethical approvals for secondary analysis.
